# The crayfish-rice coculture model contributes to regulating the soil fertility of rice fields and maintaining the stability of soil microbial community composition and function

**DOI:** 10.1007/s44307-026-00106-x

**Published:** 2026-04-28

**Authors:** Dongdong Wei, Chengguang Xing, Shenzheng Zeng, Dongwei Hou, Zhixuan Deng, Xinghai Long, Hao Wang, Renjun Zhou, Lingfei Yu, Nana Shu, Zhonghu Tao, Xi Zhou, Shaoping Weng, Jianguo He, Zhijian Huang

**Affiliations:** 1 State Key Laboratory of Biocontrol, Southern Marine Sciences and Engineering Guangdong Laboratory (Zhuhai), School of Agriculture and Biotechnology, Sun Yat-Sen University, Shenzhen, China; 2 School of Life Sciences, Sun Yat-Sen University, Guangzhou, China; 3 Guangxi Academy of Marine Sciences, Guangxi Academy of Sciences, Nanning, China; 4 Qianjiang Crayfish Industry Development and Promotion Center, Qianjiang, China

**Keywords:** Rice-crayfish coculture, Soil fertility, Microbial community stability, Community assembly, Co‑occurrence network, Functional metagenomics

## Abstract

Rice-fish coculture represents a classic sustainable agricultural paradigm; however, the microecological mechanisms underlying its capacity to maintain soil fertility and microbial community stability remain poorly understood. We conducted a 13-month field experiment comparing three cultivation systems:crayfish-rice coculture (CRCE), crayfish-waterweed coculture (CWCE), and rice monoculture (RME)-by integrating physicochemical analysis, 16S rRNA sequencing, metagenomics, microbial network analysis, and null model simulations. Our results demonstrated that coculture systems, particularly CRCE, enhanced soil fertility through carbon sequestration (total carbon: 25.0–45.0 mg/g; total organic carbon: 15.0–35.0 mg/g) and sustained redox homeostasis (consistently low oxidation–reduction potential: − 150 to − 50 mV), in stark contrast to the extreme redox fluctuations observed in RME. These stable edaphic conditions imposed deterministic selection on microbial communities (homogeneous selection contribution: 30%–50% in CRCE vs. 10%–20% in RME), shifting community assembly from stochastic drift dominance toward predictable succession. This assembly shift enriched functionally coupled keystone taxa, including iron reducers (*Geobacter*), sulfur oxidizers (*Sulfuricurvum*), and nitrifiers (*Nitrospira*), which formed ecological networks characterized by 98.6% positive interactions and enhanced functional gene repertoires associated with carbon, nitrogen, and sulfur biogeochemical cycles. Metagenomic analysis corroborated these findings, revealing enrichment of functional genes involved in polymer degradation, nitrification, and sulfate reduction in CRCE, supporting enhanced nutrient cycling capacity. We establish a hierarchical causal pathway in which bioturbation-induced environmental stabilization drives deterministic community assembly, which in turn promotes keystone taxon enrichment and functional integration. This framework provides a mechanistic explanation for how crayfish-rice coculture regulates soil fertility and sustains microbial community compositional and functional stability in anthropogenically designed agricultural ecosystems.

**Supplementary Information:**

The online version contains supplementary material available at 10.1007/s44307-026-00106-x.

## Introduction

Global demands for food security and environmental pressures exerted by intensive agriculture, including ecosystem service degradation and non-point source pollution, have created a profound tension, rendering sustainable farming models that simultaneously maintain soil fertility and microbial ecosystem stability an urgent priority. Rice-fish coculture is widely recognized as a classic paradigm for agricultural ecological intensification (Cui et al. 2023). However, the microbial mechanisms underlying its capacity to regulate soil fertility and sustain community stability remain poorly elucidated. This critical knowledge gap persists despite the model’s widespread adoption, as the microbiological processes driving the unique biogeochemical cycles, particularly those governing carbon, nitrogen, and sulfur metabolism, that underpin ecological sustainability have yet to be fully deciphered (Zhu et al. 2023). While the macroscopic ecological benefits of this model, such as reduced pesticide use, enhanced nutrient recycling, and stable yields, have been well documented (Ren et al., 2014; Xie et al. 2011), the soil-associated processes driving these ecosystem services lack mechanistic clarification. A critical scientific question therefore persists: does coculture merely alter the physicochemical properties of soils, or can it actively reshape microbial community assembly and functional gene architecture to construct a self-sustaining and stable microecosystem that underpins both soil fertility and community functional stability?

Previous studies have validated the agroecological value of rice-fish coculture systems from multiple perspectives. For instance, rice-fish systems can reduce pesticide application by 68% (Xie et al. 2011) and improve nitrogen use efficiency by 24% through complementary nutrient utilization (Cui et al. 2023). Traditional rice–common carp coculture maintains higher genetic diversity than intensive monoculture, thereby mitigating inbreeding depression. The shading effect of the rice canopy lowers water temperature by 2–3 °C, effectively buffering heat stress on aquatic animals (Hu et al. 2016). Additionally, fish bioturbation increases soil redox potential by 15–20%, accelerating organic matter turnover (Yang et al., 2017). These macroscopic advantages have positioned rice-fish coculture as a flagship of global sustainable agriculture, with the Food and Agriculture Organization (FAO, 2022), designating it a Globally Important Agricultural Heritage System. Yet, the microbial underpinnings of these benefits-particularly those related to soil fertility maintenance and community stability-remain largely unexplored.

Coculture systems are not static land-use types but dynamic ecosystems undergoing continuous biological engineering. As an economically valuable aquatic animal, the crayfish (*Procambarus clarkii*) not only inhabits paddy fields but also physically and chemically restructures the soilary environment through burrowing (up to 50 cm depth) (Dorn and Wojdak, 2004) and detritus feeding (accounting for 62% ± 7% of its diet) (Barbaresi et al., 2004). Its companion plants-whether rice or waterweed-further modulate microenvironmental conditions via rhizosphere effects and canopy shading (Hu et al. 2016). These biotic factors interact synergistically, with the soil microbiome serving as both a sensor and effector of these interactions. For instance, emerging evidence indicates that the microbiome in integrated systems primarily converts inorganic nitrogen into organic forms through assimilatory/dissimilatory nitrate reduction rather than through pollutant-generating denitrification, providing a genetic basis for enhanced nitrogen assimilation capacity (Zhang et al. 2022). It follows that the capacity of coculture systems to regulate soil fertility and maintain ecosystem stability is largely contingent upon the stability of their soil microbiome. Nevertheless, direct empirical evidence supporting this theoretical inference remains scarce, particularly studies that integrate long-term temporal dynamics, functional potential, and network-level interactions. The long-term ecological effects and microbial mechanisms underlying more complex polyculture models (e.g., rice-crayfish-turtle) on soil health and crop productivity also remain to be systematically elucidated through long-term experiments (Li et al. 2022).

Existing research on crayfish-rice coculture has predominantly relied on single-time-point sampling and 16S rRNA gene amplicon sequencing. Our previous comparative study also revealed significant differences in soil bacterial community composition between coculture and monoculture systems (Wei et al. 2021). While these efforts have documented compositional shifts in soil bacterial communities (Zhang et al. 2024), single-time-point sampling cannot distinguish transient environmental fluctuations from systematic regime shifts-precluding assessments of community stability and resilience, which manifest only over successional timescales. Whether changes in community composition translate into alterations in metabolic potential relevant to soil fertility remains unknown. Furthermore, microbial communities function through complex interaction networks, yet how coculture restructures the topological architecture of these networks to enhance functional stability remains largely unexplored. Collectively, these research gaps obscure the causal pathway linking crayfish bioturbation effects to soil microbiome stability and its fertility-regulating capacity.

To address these gaps, we conducted a 13-month field experiment in Qianjiang, Hubei Province-a core region of crayfish coculture in China. Monthly soil samples were systematically collected from six crayfish-rice coculture (CRCE), three crayfish-waterweed coculture (CWCE), and three rice monoculture (RME) systems. Nine environmental physicochemical parameters were measured, and bacterial community dynamics were characterized using 16S rRNA gene amplicon sequencing. Additionally, metagenomic sequencing was performed on soil samples from CRCE and RME systems to resolve the functional gene repertoires governing carbon, nitrogen, phosphorus, and sulfur biogeochemical cycles-key processes underpinning soil fertility. To address these gaps, we formulated three interrelated scientific questions. (i) Does crayfish-plant coculture drive the soil microbiome toward a more stable successional trajectory, thereby maintaining community compositional stability, and do different plant types (rice vs. waterweed) exert differential stabilizing effects? (ii) Does coculture enrich the functional gene repertoires of the soil microbiome and enhance functional coupling among different elemental cycling pathways, thereby regulating soil fertility potential? (iii) Can we identify and validate a hierarchical causal pathway: from “crayfish bioturbation effects, environmental filtering, keystone taxa selection, interaction network stabilization, functional gene enrichment for fertility maintenance”? By integrating time-series analysis, network ecology, and functional metagenomics, this study aims to provide a mechanistic explanation for how an anthropogenically designed agricultural ecosystem achieves soil microbiome stability and regulates soil fertility-advancing our understanding of sustainable agriculture from macroscopic observations to microbial mechanisms.

## Materials and methods

### Study site description and soil sampling

**Fig. 1:**
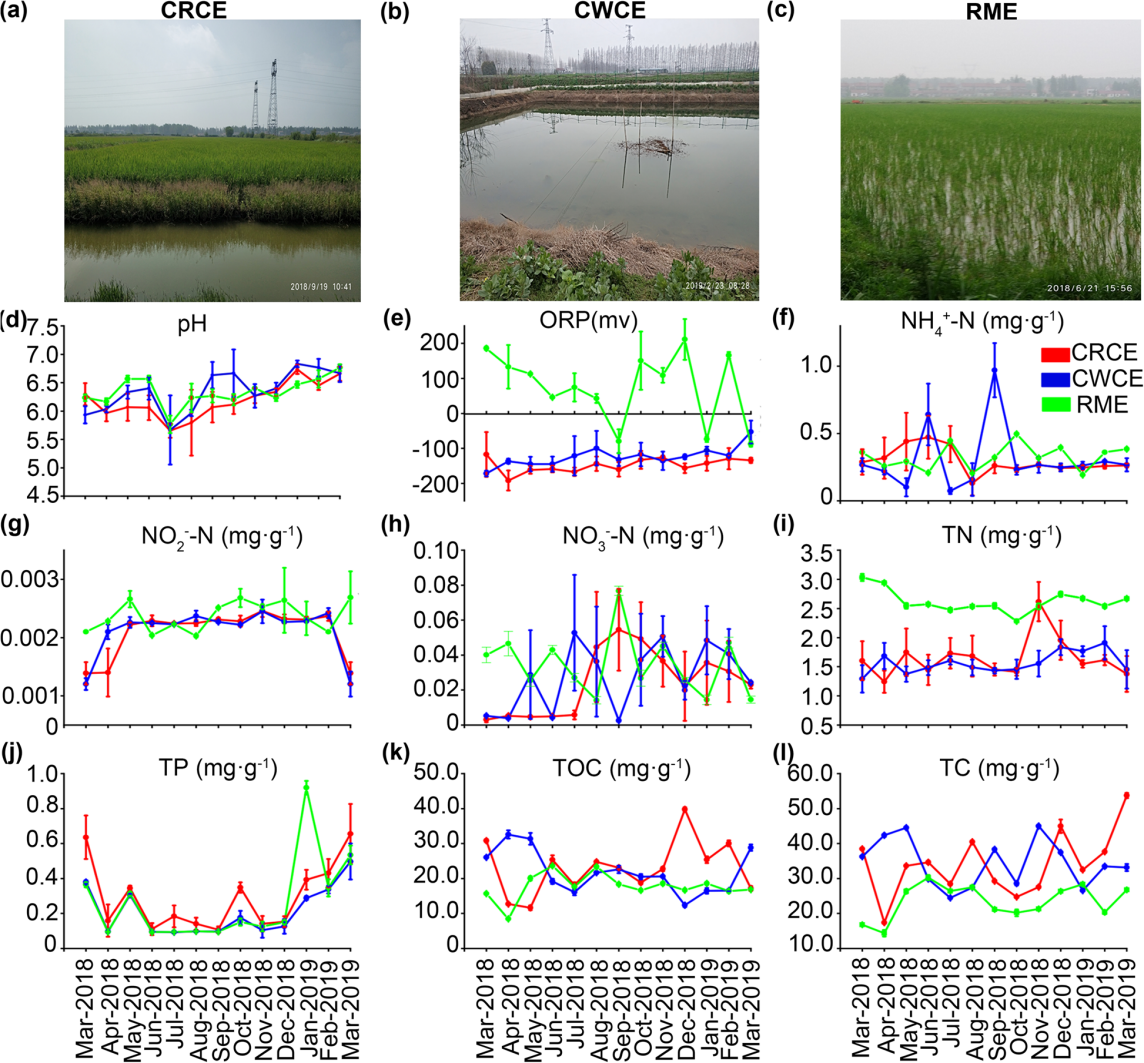
Sampling sites of the crayfish-rice coculture ecosystem (CRCE, **a**), crayfish-waterweed coculture ecosystem (CWCE, **b**) and rice monoculture ecosystem (RME, **c**). Temporal dynamics of physicochemical factors from March 2018 to March 2019, including pH (**d**), oxidation-reduction potential (ORP, **e**), NH₄⁺-N (**f**), NO₂⁻-N (**g**), NO₃⁻-N (**h**), total nitrogen (TN, **i**), total phosphorus (TP, **j**), total organic carbon (TOC, **k**), total carbon (TC, **l**). Colored broken lines represent different treatment groups: red for CRCE, blue for CWCE, and green for RME. Data points indicate measured values, and error bars represent the standard deviation

This study was conducted at the Qianjiang Crayfish-Rice Farming Base (112.48° E, 30.07° N) in Qianjiang City, Hubei Province, China. The region experiences a subtropical monsoon climate, with a mean annual temperature of 16.1℃ and mean annual precipitation of 1,100 mm. Six CRCE ponds (Fig. 1a), three CWCE ponds (Fig. 1b), and three RME paddies (Fig. 1c) were selected for this study.

The CRCE system (total area 5.33 ha) consisted of a central rice paddy surrounded by engineered peripheral ditches (2 m width × 1.5 m depth), which supported the culture of *Procambarus clarkii*. The CWCE system (0.33 ha each) maintained an average water depth of 1.5 m, with approximately 20% of the water surface covered by *Elodea nuttallii*. Juvenile crayfish (mean initial body length 0.8 cm) were stocked at densities of 10,000 ind. ha⁻^1^ in CRCE and 100,000 ind.ha⁻^1^ in CWCE. Commercial feed was administered daily after 16:00 from March to June, triweekly from July to October, and withheld from November to February.

Soil sampling was performed monthly from March 2018 to March 2019. At each sampling event, surface soil (0–5 cm depth) was collected from five randomly selected plots per pond or paddy using stainless steel corers (5 cm diameter). Soil cores from the same plot were homogenized to form one composite sample, yielding a total of 156 samples (12 sites × 13 months). Samples were immediately transported to the laboratory on ice, sieved through a 2‑mm mesh to remove plant debris and aliquoted for physicochemical analyses and molecular assays. Subsamples for DNA extraction were stored at −80 °C until further processing.

### Physicochemical analysis

Temperature (T), oxidation–reduction potential (ORP), and pH of soil samples were measured in situ (ZD-06, ZD Instrument, China). Total nitrogen (TN) and total phosphorus (TP) were measured according to previously described methods (Wei et al. 2021). Total carbon (TC) and organic carbon (TOC) were quantified using a TOC analyzer (Aurora 1030, OI Analytical, US); ammonium (NH₄⁺-N), nitrite (NO₂⁻-N), and nitrate (NO₃⁻-N) were measured by indophenol blue and spectrophotometric methods using an automated discrete analyzer (Clever Chem380, DeChemTech, Germany). Climatic variables (mean annual temperature and precipitation) for the sampling site were extracted from the WorldClim database (https://www.worldclim.org) based on geographic coordinates.

### 16S rRNA gene amplicon sequencing data analysis

Total genomic DNA was extracted from 0.5 g of each soil sample using the DNeasy PowerSoil Pro Kit (Qiagen, Germany) following the manufacturer’s instructions. The V4-V5 hypervariable region of the bacterial 16S rRNA gene was amplified using primers 338 F (5'-ACTCCTACGGGAGGCAGCAG-3') and 806R (5'-GGACTACHV GGGTWTCTAAT-3'). PCR products were purified, quantified, and pooled at equimolar concentrations. Sequencing was performed on the Illumina NovaSeq 6000 platform (PE250) at Meiji Bioinformatics Technology Co., Ltd. (Shanghai, China).

Raw paired-end reads were assembled using FLASH v1.2.7 to generate full-length sequences (Magoc and Salzberg 2011). The Quantitative Insights Into Microbial Ecology (QIIME) pipeline (v1.7.0) was employed for quality filtering of raw tags, removing low-quality regions and retaining high-quality clean tags. Chimeric sequences were subsequently identified and removed via UCHIME (v8.0.1517) by comparison with the Gold database, yielding effective reads (Edgar et al. 2011). Operational taxonomic units (OTUs) were clustered at 97% sequence similarity using the UPARSE pipeline (Edgar 2013). Representative sequences of each OTU were taxonomically annotated with the ribosomal database project (RDP) classifier (v2.2) at an 80% confidence threshold, referencing the SILVA 138 database (Wang et al. 2007). Multiple sequence alignment was performed using MUSCLE to construct a phylogenetic tree for OTUs (Edgar 2004). To mitigate sequencing depth bias, all samples were rarefied to the minimum sequencing depth, generating a standardized OTU abundance matrix.

Alpha diversity indices (Chao1, Shannon) were calculated using QIIME. Beta diversity was visualized by principal coordinate analysis (PCoA) based on Bray–Curtis distance matrices to visualize bacterial community structural differences among the three systems (Oksanen et al. 2013). Analysis of similarity (ANOSIM) with 999 permutations was applied to assess inter-group dissimilarity (Warton et al. 2012). A random forest algorithm (500 decision trees) (Zhang et al. 2018) identified discriminatory taxa in soils under CRCE and CWCE modes. The Inferring Community Assembly Mechanisms by Phylogenetic bin-based null model analysis (iCAMP) framework quantified deterministic and stochastic processes governing soil community assembly in all three systems (Ning et al. 2020). For iCAMP analysis, phylogenetic bins were defined based on the phylogenetic tree constructed from representative OTU sequences using the pheatmap and iCAMP packages in R. The phylogenetic signal was assessed using the 'ps' parameter, which was set to 0.2 based on the optimal value determined by the iCAMP package's built-in function. The null model analysis was performed with 1,000 randomizations to ensure robust estimation of the relative contributions of selection, dispersal, and drift processes. Bins with fewer than 20 OTUs were excluded from the analysis following the package recommendations (Zhou and Ning 2017). Redundancy Analysis (RDA) combined with Monte Carlo permutation tests (999 permutations) elucidated associations between soil physicochemical factors and bacterial communities. Non-metric species co-occurrence networks were constructed using the SparCC algorithm to characterize bacterial interaction patterns in CRCE, CWCE, and RME soils (Bastian et al. 2009). The correlation threshold of ρ > 0.6 was selected based on the random matrix theory (RMT) approach, which automatically determines the appropriate threshold by identifying the transition point between random and non-random associations in the correlation matrix. The *P*-value threshold of 0.01 was applied after false discovery rate (FDR) correction to minimize spurious associations (Leps and Smilauer 2006).

### Metagenomic sequencing and analysis of soil microbiomes

PE library construction was performed using the NEXTFLEX Rapid DNA-Seq Kit (Bioo Scientific, USA). The main steps included adapter ligation, magnetic bead selection to remove adapter self-ligated fragments, PCR amplification to enrich the library template, and magnetic bead recovery of PCR products to obtain the final library. Subsequently, metagenomic sequencing was conducted by Shanghai Majorbio Bio-Pharm Technology Co., Ltd. on the Illumina NovaSeq™ X Plus platform (Illumina, USA). The template DNA sequence was ultimately determined based on the collected fluorescence signals from each cycle. After obtaining raw data, fastp software (version 0.20.0, https://github.com/OpenGene/fastp) was used to trim adapter sequences from the 3' and 5' ends of reads. Reads shorter than 50 bp or with an average base quality score below 20 after trimming were removed, retaining high-quality sequences. For samples originating from a host with a published genome sequence, reads were aligned to the host DNA sequence using BWA software (version 0.7.17, http://bio-bwa.sourceforge.net). Reads showing high alignment similarity, considered potential contamination, were removed. The optimized sequences were assembled using MEGAHIT software (version 1.1.2, https://github.com/voutcn/megahit). (Specific assembly methods can be determined based on project requirements by contacting technical support). Contigs with a length of ≥ 300 bp were selected from the assembly results as the final assembly output. Open reading frames (ORFs) within these contigs were predicted using Prodigal software (version 2.6.3, https://github.com/hyattpd/Pr odigal). Genes with a nucleic acid length of ≥ 100 bp were selected and translated into amino acid sequences (Li et al. 2016).

To construct a non-redundant gene catalog, CD-HIT software (version 4.7, http://weizhongli-lab.org/cd-hit/) was used to cluster all predicted gene sequences from all samples (parameters: 90% identity, 90% coverage). The longest sequence from each cluster was selected as the representative sequence. Subsequently, SOAPaligner software (version soap2.21 release, https://github.com/ShujiaHuang/SOAPaligner) was employed to align high-quality reads from each sample against the non-redundant gene catalog (95% identity) to determine the abundance of each gene in the corresponding sample (Kanehisa et al. 2012). For taxonomic and functional annotation, Diamond software (version 2.0.13, https://github.com/bbuchfink/diamond) was used to perform BLASTP alignments (e-value ≤ 1e-5) of the amino acid sequences from the non-redundant gene catalog against the NR database (Gemayel et al. 2022) and the KEGG database, respectively (Smith et al. 2005). Taxonomic annotation was obtained using the taxonomy information database associated with the NR database (Parks et al. 2015). Species abundance was calculated as the sum of the abundances of genes assigned to that species. Based on the KEGG annotation results, the abundance for functional categories such as KO, Pathway, EC, and Module was calculated as the sum of the abundances of genes assigned to that specific functional category (Rühlemann et al. 2022).

### Statistical analysis

All statistical analyses were performed using R software (version 4.0.2). For 16S rRNA gene amplicon sequencing data, alpha diversity indices (Shannon and Chao1) were compared among CRCE, CWCE, and RME using Welch's two-sample t-test, with significance thresholds set at *P* < 0.05 and *P* < 0.01 (Hoffman 2019). Beta diversity was visualized via principal coordinate analysis (PCoA) based on Bray–Curtis dissimilarity matrices. Permutational multivariate analysis of variance (PERMANOVA) with 999 permutations was applied to test for significant differences in bacterial community structure among habitat types, and Analysis of similarities (ANOSIM) was additionally used to assess inter-group dissimilarity. Differentially abundant bacterial genera between habitat pairs were identified using the Wilcoxon rank-sum test. Redundancy analysis (RDA) was conducted to investigate relationships between bacterial community structure and environmental factors, with significance tested using Monte Carlo permutation tests (999 permutations). Spearman's rank correlation coefficients were calculated to examine associations between specific environmental factors and bacterial community composition (Myers and Sirois 2006). For metagenomic functional gene data, alpha diversity indices of functional genes were compared between CRCE and RME using Welch's t-test. Differences in overall functional gene composition between the two habitats were assessed by PERMANOVA based on Bray–Curtis distances. Differentially abundant functional genes between CRCE and RME were identified using the Wilcoxon rank-sum test with false discovery rate (FDR) correction. Mantel tests were performed to examine correlations between environmental factor matrices and functional gene composition matrices, with significance levels denoted as *P* < 0.05 and *P* < 0.01. All data were presented as mean ± standard deviation, and all statistical tests were two-sided.

## Results

### Coculture systems reshape the soil physicochemical environment

During the 13-month sampling period, pH (Fig. 1d) and ORP (Fig. 1e) exhibited distinct patterns across CWCE, CRCE, and RME. CWCE maintained relatively stable pH levels (5.5–7.0), with slight fluctuations (6.2 in Apr-2018 and 5.8 in Nov-2018). In contrast, CRCE showed slightly lower pH values (5.0–6.5), reaching a minimum of 5.2 in Oct-2018. RME exhibited greater variability (5.5–7.5), peaking at 7.2 in Jul-2018 before declining to 5.7 in Dec-2018. ORP trends also differed significantly: CRCE remained consistently low (− 150 to − 50 mV), CWCE was more stable (− 50 to 50 mV), while RME displayed extreme variability (< − 100 to > 200 mV), with notable peaks in May-2018 (200 mV) and Jan-2019 (− 150 mV). CRCE showed variable NH₄⁺-N levels (0.2–0.8 mg/g), peaking in Jul-2018 (0.8 mg/g) and reaching a minimum in Nov-2018 (0.2 mg/g). CWCE displayed greater NH₄⁺-N fluctuations (0.1–1.0 mg/g), with an Aug-2018 maximum (1.0 mg/g) and Dec-2018 minimum (0.1 mg/g), while RME maintained lower but variable concentrations (0.2–0.6 mg/g), peaking in Jun-2018 (0.5 mg/g) (Fig. 1f). RME showed minor NO₂⁻-N concentrations fluctuations (< 0.002 mg/g), with a Mar-2019 maximum, whereas CWCE and CRCE maintained stable levels below 0.0015 mg/g and 0.001 mg/g, respectively (Fig. 1g). NO₃⁻-N exhibited the greatest variability in RME (0–0.08 mg/g), with a Nov-2018 peak (0.07 mg/g). In contrast, CWCE and CRCE demonstrated lower and more stable concentrations, generally remaining below 0.04 mg/g and 0.03 mg/g, respectively (Fig. 1h). CRCE exhibited lower TN (0.8–1.6 mg/g) compared to CWCE (1.0–2.0 mg/g), with a peak of 1.8 mg/g in CWCE during Aug-2018. In CRCE, TN was approximately 1.2 mg/g in Mar-2019. RME showed relatively stable TN levels initially, but concentrations increased towards the end of the sampling period, reaching 3.0 mg/g in Mar-2019 (Fig. 1i). TP in CRCE ranged from 0.05 to 0.5 mg/g, peaking at 0.45 mg/g in Dec-2018. CWCE had lower TP levels (0.1–0.4 mg/g) than RME, which varied from 0.05 mg/g (Apr-2018) to 0.8 mg/g (Feb-2019) (Fig. 1j). TOC in CRCE ranged from 15.0 to 35.0 mg/g, with a peak of 32.0 mg/g in Nov-2018. CWCE showed fluctuations between 10.0 and 30.0 mg/g, peaking at 28.0 mg/g in Oct-2018. RME had lower average TOC levels (5.0–20.0 mg/g), with a maximum of 18.0 mg/g in Jul-2018 (Fig. 1k). TC in CWCE varied between 20.0 and 40.0 mg/g, reaching 35.0 mg/g in Aug-2018. CRCE exhibited similar TC levels (25.0–45.0 mg/g), with 30.0 mg/g recorded in Jan-2019. RME had lower TC concentrations (10.0–30.0 mg/g), peaking at 28.0 mg/g in Sep-2018 (Fig. 1l). Taken together, compared with RME, CRCE significantly increased soil TC and TOC contents- clear indicators of enhanced organic matter accumulation and improved soil fertility. Furthermore, CRCE maintained consistently stable ORP across the 13-month experimental period, which stood in stark contrast to the extreme ORP fluctuations observed in RME. These results demonstrate that CRCE simultaneously boosts soil fertility (via carbon sequestration) and reinforces environmental stability (via redox buffering), two interconnected yet distinct dimensions of soil health that collectively underpin the long-term sustainability of the agricultural system.

### Coculture alters soil microbial community structure and composition

**Fig. 2:**
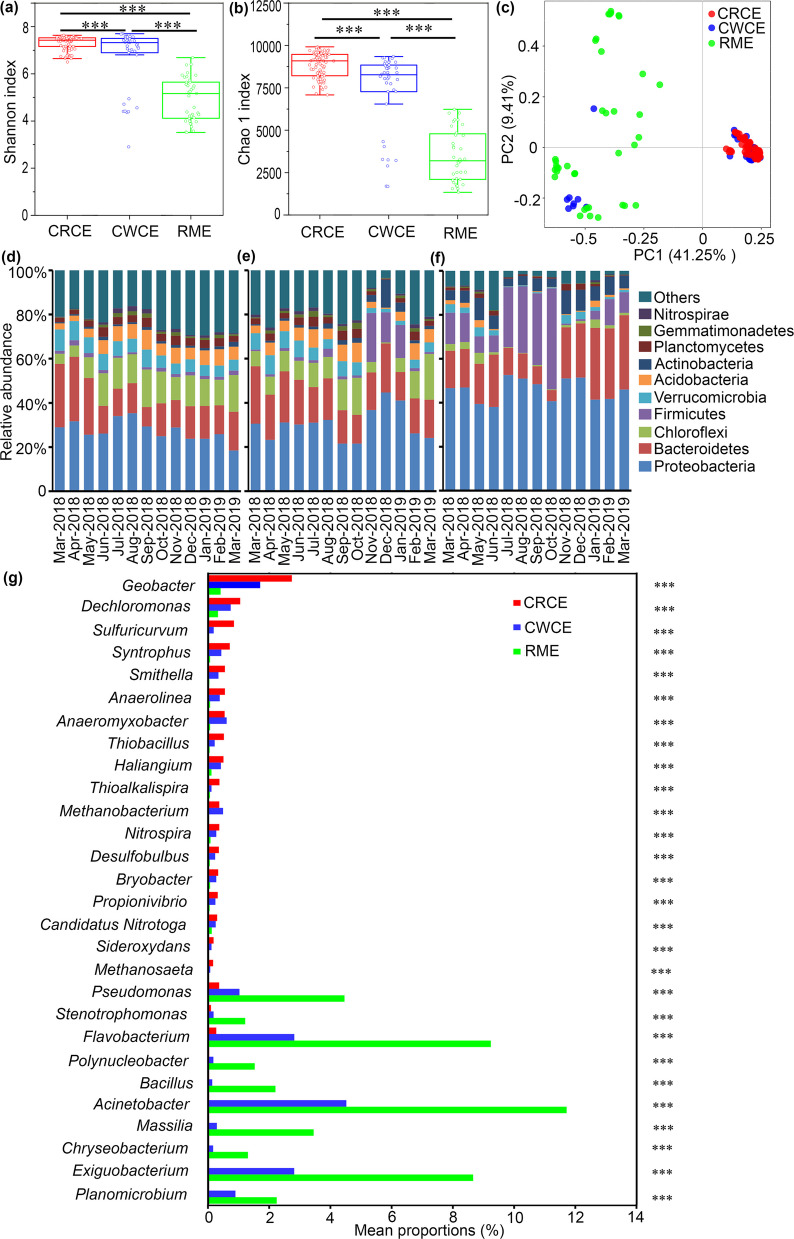
Diversity, community composition, and distinctive species analyses based on 16S rRNA gene sequencing. Statistical comparisons of the Shannon index (**a**) and Chao1 index (**b**) among CRCE, CWCE, and RME habitats using Welch’s *t*-test (*P* < 0.05; *P* < 0.01). PCoA of soil bacterial community structure based on the Bray–Curtis distances (**c**). Relative abundance of bacterial phyla in CRCE (**d**), CWCE (**e**) and RME (**f**) across the 13-month sampling period, each bar represents the mean of triplicate samples from each time point. Differentially abundant bacterial genera identified by Wilcoxon rank-sum test (**g**)

Based on 16S rRNA gene amplicon sequencing, the alpha and beta diversity of soil microbial communities were compared among CRCE, CWCE, and RME systems. The Shannon index (Fig. 2a), was highest in CRCE soils (6.5 ± 0.3), followed by CWCE (5.8 ± 0.4), and lowest in RME (5.2 ± 0.5); the Chao1 index (Fig. 2b) showed that CRCE had the highest species richness (2,850 ± 120), CWCE intermediate (2,450 ± 150), and RME the lowest (2,100 ± 180), indicating that coculture systems, particularly CRCE, support greater microbial diversity and species richness compared to rice monoculture. PCoA based on Bray–Curtis distances demonstrated distinct clustering of microbial communities, with the first two principal coordinates explaining 41.25% and 9.41% of the total variation, respectively (Fig. 2c). ANOSIM confirmed significant differences among the three groups (R = 0.588, *P* < 0.001), demonstrating that cropping system exerts a strong deterministic effect on soil microbial community structure.

In CWCE (Fig. 2d), Proteobacteria was one of the dominant phyla, exhibiting significant temporal dynamics in relative abundance, ranging from 21.584% (Jul-2018) to 44.692% (Oct-2018). For instance, in the first month of sampling, its relative abundance was 30.558%. Bacteroidetes also displayed temporal variations, with a relatively low abundance of 20.339% in Apr-2018, increasing to 28.736% by Dec-2018. Chloroflexi showed notable variability, peaking at 43.600% in Mar-2019 and reaching its lowest level (20.658%) in Oct-2018. Additionally, Firmicutes exhibited higher relative abundance (22.210%) in Sep-2018, while remaining relatively low in most other months. In CRCE (Fig. 2e), Proteobacteria remained one of the predominant phyla, with relative abundances fluctuating between 23.775% and 35.355%. For example, in May-2018, its abundance reached 35.355%. Bacteroidetes abundance ranged from 8.77% to 29.184%, with the lowest value observed in Oct-2018 and a higher value (25.735%) in Feb-2019. Chloroflexi abundance also varied across months, from 5.097% in Mar-2018 to 14.743% in Jul-2018. Compared to CWCE samples, CRCE samples exhibited distinct patterns in phylum-level abundance dynamics. RME samples displayed unique microbial community compositions (Fig. 2f). Proteobacteria dominated in most months, with relative abundances ranging from 18.452% (Apr-2018) to 52.617% (May-2018), such as 46.908% in May-2018. Notably, Firmicutes exhibited significantly higher abundance in RME samples, reaching 27.312% in Jul-2018 and peaking at 45.912% in Dec-2018, which contrasted sharply with CWCE and CRCE samples. Bacteroidetes abundance was highest (33.82%) in Mar-2019. The soil microbial communities in CWCE, CRCE, and RME exhibited significant temporal variations and inter-habitat differences in taxonomic composition. These variations may reflect distinct environmental conditions, such as redox potential and pH, influencing microbial community structure and function in these ecosystems.

### Keystone microbial taxa responsive to different coculture practices

Differentially abundant bacterial genera were identified using the Wilcoxon rank-sum test with false discovery rate (FDR) correction. Taxa with log2 fold change > 1 and FDR-adjusted *P* < 0.05 were considered significantly enriched. The results showed distinct differences in the relative abundances of various taxa among the three systems (Fig. 2g). For *Geobacter*, the relative abundances in CWCE, CRCE and RME were 1.70%, 2.73% and 0.40% respectively. Its higher abundance in CRCE suggests that the environmental niche in CRCE may be more favorable for *Geobacter* proliferation compared to CWCE and RME. *Dechloromonas* also showed habitat-specific abundance patterns, with higher abundance in CRCE (1.05%) than in CWCE (0.74%) and RME (0.32%). This indicates that CRCE may provide a more suitable ecological niche for *Dechloromonas*. *Sulfuricurvum* exhibited significantly higher abundance in CRCE (0.847%), while its abundance was much lower in CWCE (0.18%) and RME (0.03%). This suggests that environmental factors such as nutrient availability and ORP in CRCE may promote its growth. Other taxa, including *Syntrophus*, *Smithella*, *Anaerolinea*, *Anaeromyxobacter*, *Thiobacillus*, *Haliangium, Thioalkalispira*, *Methanobacterium*, *Nitrospira*, *Desulfobulbus*, *Bryobacter*, *Propionivibrio*, *Candidatus Nitrotoga* and *Sideroxydans,* showed significant inter-habitat variations in abundances. Generally, these taxa were more abundant in CRCE and CWCE than in RME, likely due to differences in soil environmental conditions and microbial interaction networks. In contrast, some taxa were uniquely enriched in RME. For example, *Pseudomonas* had an extremely high abundance in RME (4.46%), compared to only 1.03% in CWCE and 0.36% in CRCE. Similarly, *Stenotrophomonas*, *Flavobacterium*, *Polynucleobacter*, *Bacillus, Acinetobacter, Massilia*, *Chryseobacterium*, *Exiguobacterium*, and *Planomicrobium* were significantly overrepresented in RME (Fig. 2g). These findings suggest that RME harbors a distinct microbial community structure, possibly driven by unique environmental filtering.

### Coculture enhances the complexity and stability of microbial co-occurrence networks

**Fig. 3:**
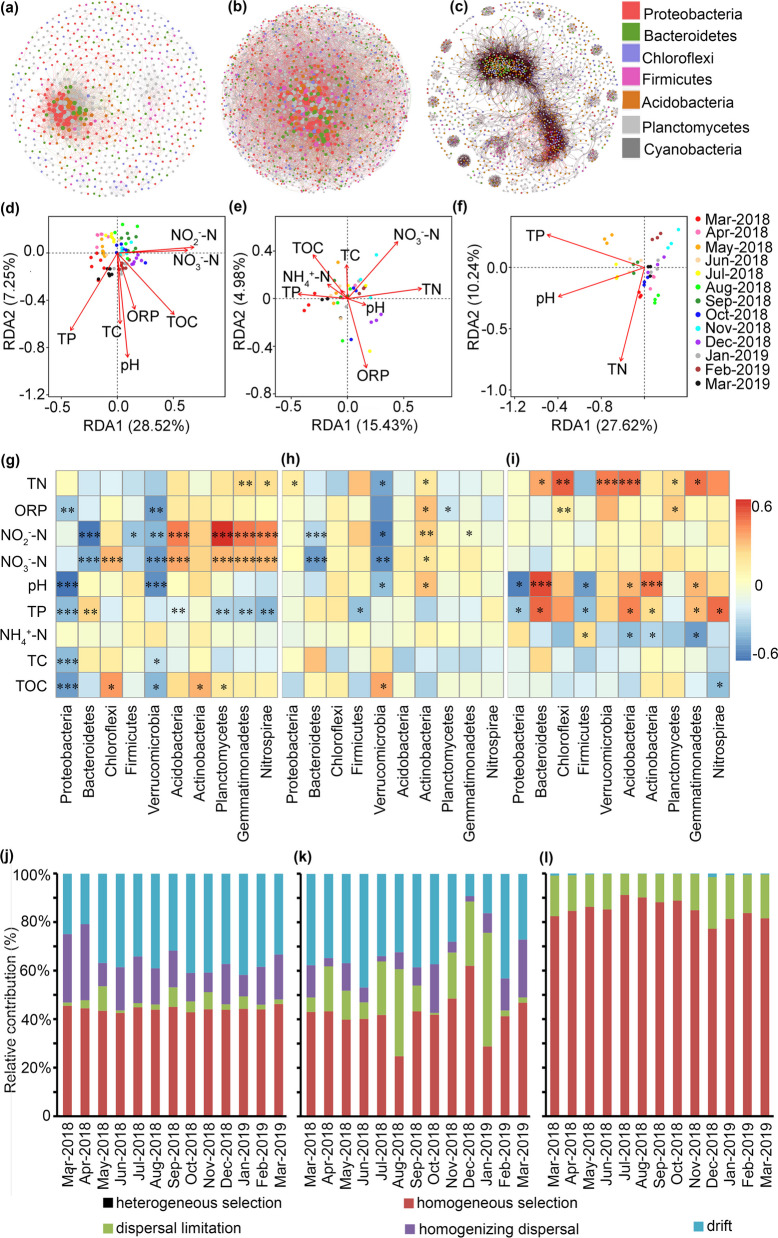
Interspecies interaction networks of the soil bacterial community in CRCE (**a**), CWCE (**b**) and RME (**c**). Each node represents a bacterial genus, with node colors corresponding to different major phyla. Edges indicate interactions: red for positive and green for negative. RDA of the relationships between soil bacterial community structure and environmental factors in CRCE (**d**), CWCE (**e**) and RME (**f**). Spearman’s correlation heatmaps between environmental factors and bacterial communities in CRCE (**g**), CWCE (**h**) and RME (**i**). The relative contributions of different ecological processes to microbial community assembly in CRCE (**j**), CWCE (**k**), and RME (**l**)

The bacterial co-occurrence networks exhibited distinct structural characteristics across the three systems. CRCE (Fig. 3a) displayed the highest complexity with 1,625 nodes and 7,986 edges, dominated by positive interactions (98.6%). CWCE (Fig. 3b) had a simpler network (694 nodes, 3,590 edges) with a higher proportion of negative interactions (16.1%). In contrast, RME (Fig. 3c) showed the highest connectivity (1,428 nodes, 20,684 edges), with 97.8% positive edges, suggesting a highly interconnected microbial community. Proteobacteria was the most abundant phylum in all networks, forming central hubs in CRCE and RME. CWCE exhibited greater taxonomic diversity, with Bacteroidetes playing a more prominent role. RME also featured significant contributions from Acidobacteria and Planctomycetes, indicating a broader microbial consortium. CRCE and RME were characterized by strong positive co-occurrences, implying mutualistic or niche-sharing relationships. CWCE, however, had more competitive interactions, possibly due to resource limitations. The dense connectivity in RME suggests functional redundancy, while the sparser CWCE network may indicate a more specialized community structure.

### Linking environmental drivers to microbial traits

In CRCE soils (Table 1), the Shannon index showed significant positive correlations with pH (R = 0.28, *P* = 0.011) and ORP (R = 0.36, *P* = 0.001), as well as with NO₂⁻-N (R = 0.562, *P* < 0.001), NO₃⁻-N (R = 0.347, *P* = 0.002), and TOC (R = 0.452, *P* < 0.001). The Chao1 index demonstrated similar trends, with positive correlations for pH (R = 0.250, *P* = 0.027), ORP (R = 0.373, *P* = 0.001), NO₂⁻-N (R = 0.446, *P* < 0.001), NO₃⁻-N (R = 0.303, *P* = 0.007), and TOC (R = 0.442, *P* < 0.001). For CWCE soils, no significant correlations were observed between microbial diversity indices (Shannon and Chao1) and any environmental factors. In RME soils, the Shannon index exhibited a significant negative correlation with NH₄⁺-N (R = −0.370, *P* = 0.020), while showing positive correlations with TN (R = 0.599, *P* < 0.001) and TP (R = 0.483, *P* = 0.002). A negative correlation was also observed with TOC (R = −0.357, *P* = 0.026). The Chao1 index demonstrated a strong positive correlation with TN (R = 0.684, *P* < 0.001).

**Table 1:** Spearman correlation analysis between environmental variables and bacterial alpha diversity across three study sites (CRCE, CWCE, RME). R values: Range from −1 (perfect negative correlation) to +1 (perfect positive correlation). Asterisk (*): Marks statistically significant correlations (*P* < 0.05)

			pH	ORP	NH_4_⁺-N	NO_2_⁻-N	NO_3_⁻-N	TN	TP	TOC	TC
CRCE	Shannon	R	0.285*	0.362*	-0.085	0.563*	0.348*	0.138	0.071	0.452*	0.134
		*P*	0.011	0.001	0.458	0.000	0.002	0.228	0.539	0.000	0.240
	Chao1	R	0.250*	0.373*	-0.090	0.447*	0.304*	0.163	-0.004	0.442*	0.212
		*P*	0.027	0.001	0.432	0.000	0.007	0.155	0.973	0.000	0.063
CWCE	Shannon	R	0.145	-0.087	0.085	0.085	0.000	-0.260	0.022	0.037	-0.151
		*P*	0.380	0.600	0.606	0.605	0.999	0.110	0.896	0.823	0.358
	Chao1	R	0.012	-0.169	0.000	0.019	-0.237	-0.349*	0.039	0.301	-0.015
		*P*	0.943	0.303	0.999	0.907	0.146	0.030	0.811	0.063	0.928
RME	Shannon	R	0.247	0.301	-0.371*	-0.035	0.195	0.600*	0.118	-0.064	-0.074
		*P*	0.130	0.063	0.020	0.832	0.234	0.000	0.475	0.697	0.656
	Chao1	R	0.247	0.318*	-0.224	0.108	0.063	0.685*	0.483*	-0.357*	-0.255
		*P*	0.129	0.049	0.170	0.511	0.703	0.000	0.002	0.026	0.118

RDA highlighted the dominant environmental filters, in CRCE (Fig. 3d), NO₃⁻-N and NO₂⁻-N were the strongest predictors (explaining 36.27% variance), with pH and ORP showing synergistic effects (RDA1 = 28.52%, RDA2 = 7.75%). In CWCE (Fig. 3e), TN, TP, and NH₄⁺-N were key drivers (20.33% variance), with pH and ORP exhibiting antagonistic effects (RDA1 = 15.43%, RDA2 = 4.90%). In RME (Fig. 3f), pH and TN dominated (29.86% variance), with TP modulating their interactions (RDA1 = 27.62%, RDA2 = 2.24%. These results collectively demonstrate that nitrogen species, pH and redox conditions are primary factors shaping microbial community assembly across soil habitats.

In CRCE, phylum-level analysis further revealed habitat-specific environmental responses (Fig. 3g), Proteobacteria in CRCE showed negative correlations with pH, TC, TOC, TP, and ORP (*P* < 0.01). Bacteroidetes was negatively correlated with NO₂⁻-N and NO_3_⁻-N (R = −0.56 and −0.41) but positively correlated with TP (R = 0.31). Chloroflexi was positively associated with TOC and NO_3_⁻-N (R = 0.47 and 0.38). Nitrogen-cycling related phyla (e.g., Acidobacteria, Planctomycetes and Nitrospirae) were positively correlated with NO₂⁻-N, NO_3_⁻-N and TN (R = 0.54–0.72). In CWCE (Fig. 3h), pH was a key driver of community composition, with Bacteroidetes and Actinobacteria showing positive correlations (R = 0.64 and 0.55), while Proteobacteria was negatively correlated (R = −0.54). Chloroflexi and Gemmatimonadetes were positively linked to TN and TP (R = 0.45, 0.60). Conversely, Firmicutes displayed negative correlations with TN and TP (R = −0.36). Notably, Nitrospirae showed a negative correlation with TOC (R = −0.36) but a positive correlation with TN (R = 0.50). RME (Fig. 3i) shared some patterns with CWCE, such as Bacteroidetes and Actinobacteria maintained strong positive correlations with pH (R = 0.64 and 0.55), Proteobacteria remained negatively correlated (R = −0.54), but also exhibited unique features, such as Verrucomicrobia's positive correlation with ORP (R = 0.41), Planctomycetes showed a negative correlation with NH₄^+^-N (R = −0.32).

In CRCE microbial communities, drift (40–60%) and homogeneous selection (30–50%) were the dominant assembly processes, while dispersal limitation (10–20%) and homogenizing dispersal (5–15%) played minor roles. Heterogeneous selection had negligible influence (< 5%). For example, in Mar-2018, drift accounted for 55% of community variation, homogeneous selection for 35%, and dispersal-related processes for ≤ 10% (Fig. 3j). CWCE communities exhibited greater temporal variability in assembly processes. Drift (30–70%) and homogeneous selection (20–50%) remained dominant but fluctuated seasonally. Dispersal limitation (10–30%) and homogenizing dispersal (5–25%) had stronger contributions than in CRCE, and heterogeneous selection occasionally reached 10%. For instance, in Aug-2018, drift (40%), homogeneous selection (30%), and dispersal limitation (20%) were key drivers (Fig. 3k). RME communities were overwhelmingly structured by drift (70–90%), with minimal contributions from homogeneous selection (10–20%). Dispersal-related processes (each < 10%) and heterogeneous selection (< 5%) had negligible effects. For example, in Dec-2018, drift explained 80% of variation, while homogeneous selection accounted for only 15% (Fig. 3l).

### Coculture drives the functional gene profiles of soil biogeochemical cycling

**Fig. 4:**
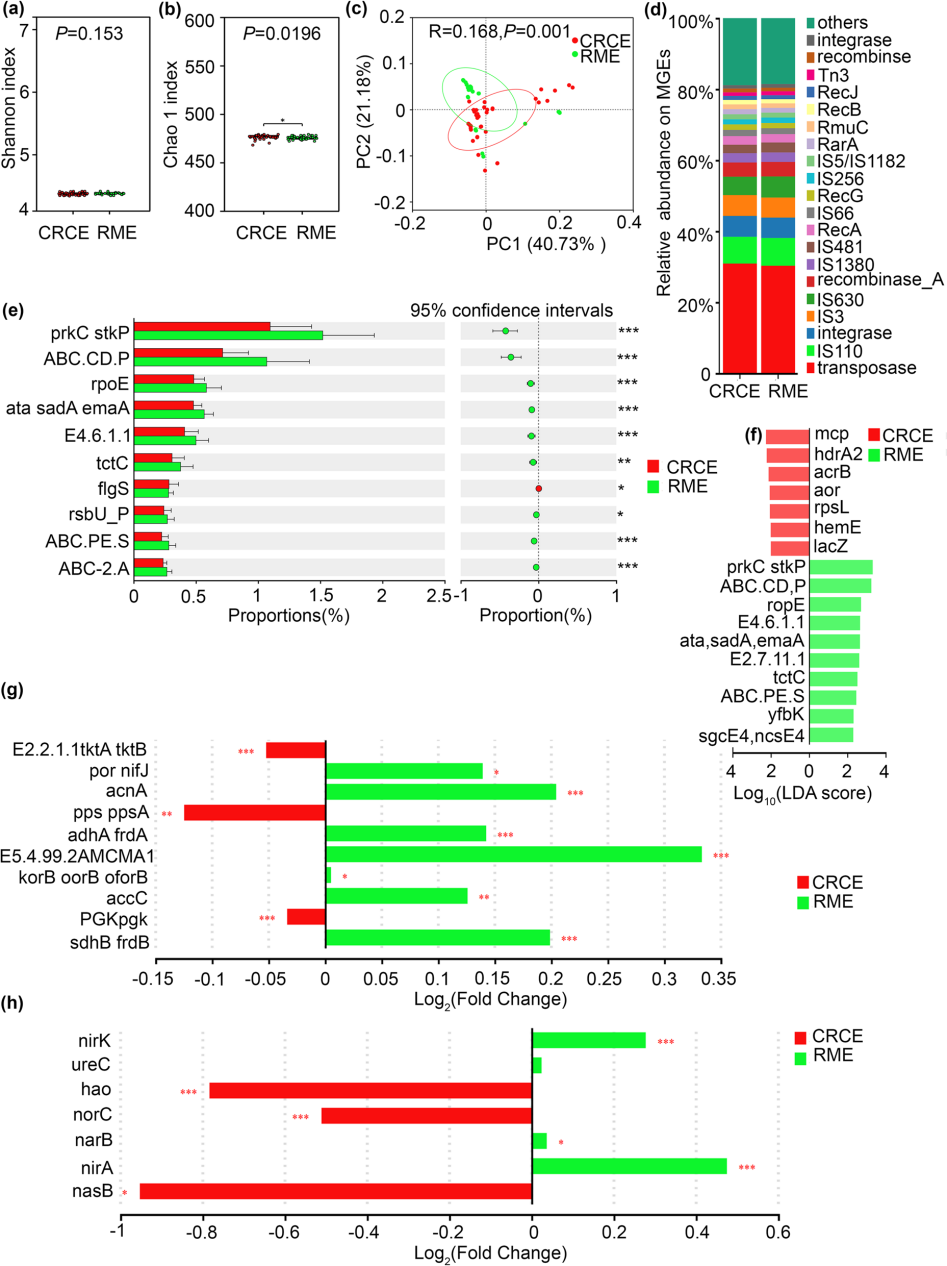
Comparison of metagenomic functional gene diversity and composition between CRCE and RME. Alpha diversity indices: Shannon index (**a**) and Chao1 index (**b**). PCoA of functional gene composition based on Bray–Curtis distances (**c**). Comparison of overall functional gene composition (**d**) and differentially abundant functional genes between CRCE and RME (**e**). Linear discriminant analysis (LDA) effect size (LEfSe) identified the most differentially abundant functional genes between CRCE and RME (**f**). Fold change of carbon cycle-related genes (**g**). Fold change of nitrogen cycle-related genes (**h**)

Metagenomic sequencing was performed on soil samples from CRCE and RME, followed by functional gene annotation based on the KEGG and MGHs databases. The results revealed that in terms of alpha diversity, no significant difference was observed in the Shannon index (Fig. 4a) between the two groups (*P* = 0.153), whereas the Chao 1 index (Fig. 4b) was significantly lower in CRCE soils than in RME soils (*P* = 0.020), suggesting similar functional gene evenness but distinct richness between the two systems. Beta diversity analysis using PCoA based on Bray–Curtis distances showed a significant separation between CRCE and RME samples (Adonis test:R = 0.168, *P* = 0.001), with the first two principal coordinates explaining 40.73% and 21.18% of the total variation, respectively (Fig. 4c). Community composition analysis based on MGHs classification indicated that the overall functional gene profiles were similar between CRCE and RME (Fig. 4d).

Differential analysis of carbon cycle-related genes in soil metagenomes under CRCE and RME systems was performed using the Wilcoxon rank-sum test (Fig. 4g). The results revealed significant differences in the abundance of multiple genes involved in key carbon metabolic pathways between the two systems. Genes associated with carbon fixation and central carbon metabolism showed contrasting enrichment patterns. The *pps*/*ppsA* gene cluster, encoding pyruvate phosphate dikinase involved in gluconeogenesis and CO₂ fixation, was significantly enriched in CRCE soils (*P* < 0.01). Similarly, the *PGK*/*pgk* gene, encoding phosphoglycerate kinase in glycolysis/gluconeogenesis, exhibited significantly higher abundance in CRCE (*P* < 0.001). The *E2.2.1.1*/*tktA*/*tktB* gene cluster, encoding transketolase in the pentose phosphate pathway, was also significantly more abundant in CRCE (*P* < 0.001). In contrast, genes involved in the tricarboxylic acid (TCA) cycle and associated respiratory processes were predominantly enriched in RME soils. The *acnA* gene (aconitase) was significantly more abundant in RME (*P* < 0.001). Both *sdhA/frdA* (*P* < 0.001) and *sdhB*/*frdB* (*P* < 0.001), encoding subunits of succinate dehydrogenase/fumarate reductase, showed significantly higher expression in RME. The *por*/*nifJ* gene cluster, involved in anaerobic pyruvate metabolism, was significantly enriched in RME (*P* < 0.05). The *korB/oorB/oforB* gene cluster, associated with anaerobic oxidative stress response and carbon metabolism, was also significantly more abundant in RME (*P* < 0.05). Notably, the *E5.4.99.2A*/*mcmA1* gene, encoding methylmalonyl-CoA mutase involved in odd-chain fatty acid and branched-chain amino acid degradation, exhibited the most pronounced differential enrichment, with significantly higher abundance in RME (*P* < 0.001). Additionally, the *accC* gene, encoding a subunit of acetyl-CoA carboxylase involved in fatty acid biosynthesis, was significantly enriched in RME (*P* < 0.01). These results demonstrate that CRCE and RME differentially influence the abundance of carbon cycle-related genes in the soil metagenome, with specific genes exhibiting significant differential enrichment under each management mode. To further elucidate the taxonomic origins of the differentially abundant carbon cycling genes identified above, we performed host tracking analysis linking functional genes to microbial genera. As shown in Fig. S1, genes involved in the reductive citrate cycle and reductive acetyl-CoA pathway were predominantly associated with *Geobacter* and *Anaeromyxobacter* in CRCE soils, while genes related to the Calvin cycle were mainly carried by *Pseudomonas* and *Bacillus* in RME soils (Fig. S1a, b). These results demonstrate that the distinct carbon cycling potentials between coculture and monoculture systems are underpinned by phylogenetically distinct microbial consortia.

Differential abundance of nitrogen cycle-related genes in soil metagenomes Differential analysis of nitrogen cycle-related genes in soil metagenomes under CRCE and RME was performed using the Wilcoxon rank-sum test (Fig. 4h). The results revealed distinct enrichment patterns for genes involved in key nitrogen transformation pathways. Genes associated with nitrification and denitrification intermediate processing were significantly enriched in CRCE soils. The *hao* gene, encoding hydroxylamine oxidoreductase involved in the second step of nitrification (conversion of hydroxylamine to nitrite), showed significantly higher abundance in CRCE (*P* < 0.001). The *norC* gene, encoding nitric oxide reductase involved in denitrification (reduction of NO to N₂O), was also significantly enriched in CRCE (*P* < 0.001). Additionally, the *nasB* gene, involved in assimilatory nitrate reduction, exhibited significantly higher abundance in CRCE (*P* < 0.05). Conversely, genes involved in denitrification and dissimilatory nitrate reduction were predominantly enriched in RME soils. The *nirK* gene, encoding copper-containing nitrite reductase catalyzing the reduction of nitrite to NO in denitrification, showed significantly higher abundance in RME (*P* < 0.001). The *nirA* gene, involved in assimilatory nitrite reduction, was also significantly enriched in RME (*P* < 0.001). The *narB* gene, encoding nitrate reductase involved in assimilatory nitrate reduction, exhibited significantly higher abundance in RME (*P* < 0.05). The *ureC* gene, encoding the alpha subunit of urease involved in urea hydrolysis, showed slightly higher abundance in RME, although the difference did not reach statistical significance. In summary, CRCE and RME differentially influence the abundance of nitrogen cycle-related genes in the soil metagenome. The significant enrichment of *hao*, *norC*, and *nasB* in CRCE, alongside the significant enrichment of *nirK*, *nirA*, and *narB* in RME, indicates that the two management systems differentially shape the genetic potential for key nitrogen transformation processes, including nitrification, denitrification, and assimilatory nitrate reduction. To assess the functional integration of biogeochemical cycling potentials, we constructed co-occurrence networks of carbon and nitrogen cycling genes. As shown in Fig. S2, CRCE exhibited more modular and tightly connected functional networks for both carbon and nitrogen cycling compared to RME, with higher modularity coefficients and shorter average path lengths (Fig. S2a, b, c, d). This enhanced network organization suggests that coculture systems foster greater metabolic coupling and functional redundancy, which are key attributes of a functionally stable ecosystem.

### Relationship between environmental factors and functional gene composition

**Fig. 5:**
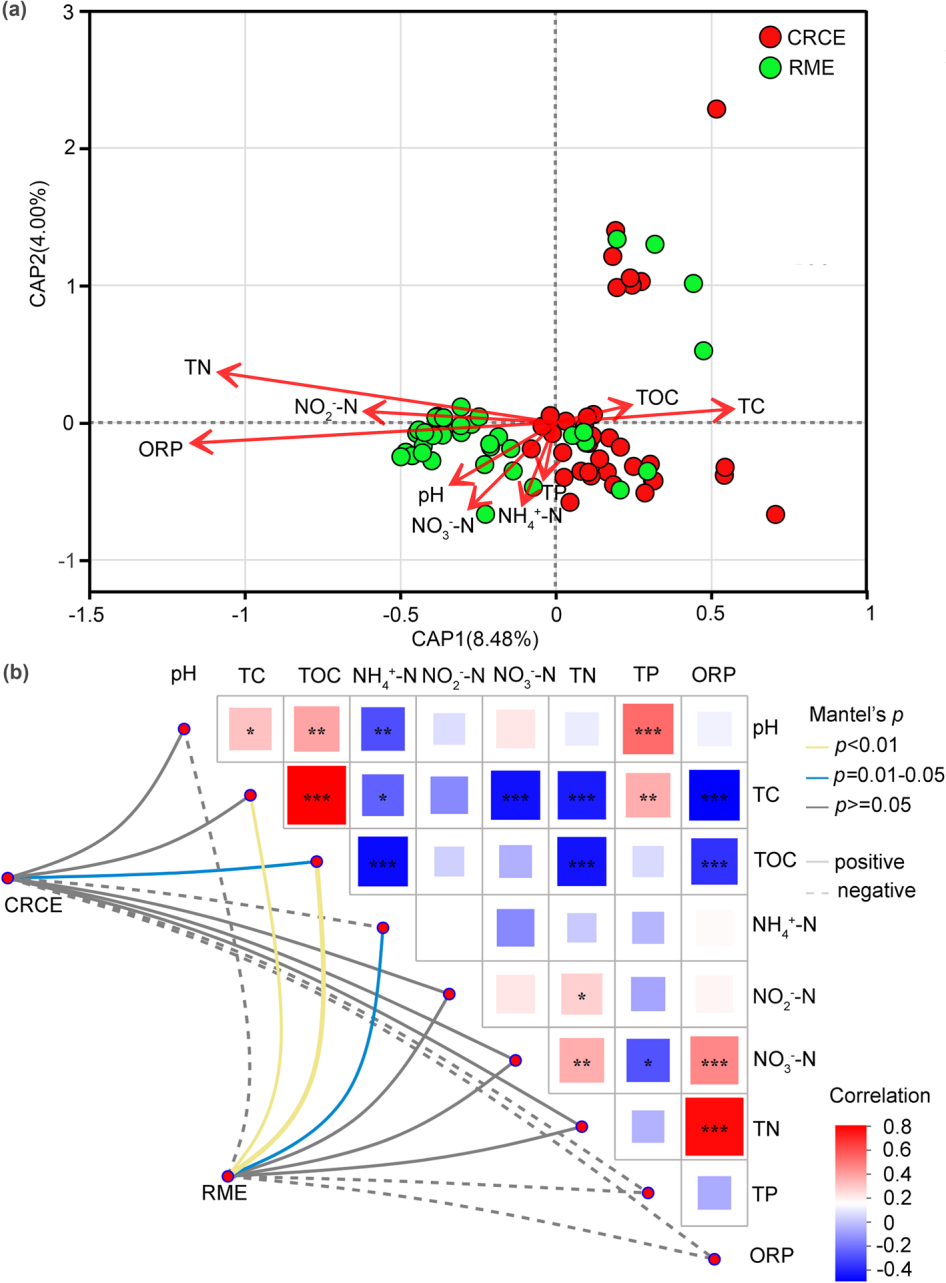
Driving effects of environmental factors on the metagenomic functional composition in CRCE and RME. RDA based on functional gene composition, showing the distribution of samples from CRCE and RME habitats with vectors indicating the direction and strength of correlations with environmental factors (**a**). Mantel test correlation network between environmental factors and major functional gene categories (**b**). Edge color indicates the direction of correlation (red for positive, blue for negative). *P* < 0.05, *P* < 0.01

RDA further revealed quantitative relationships between functional gene composition and environmental factors (CAP1: 8.48%, CAP2: 4.00%). CRCE samples exhibited dispersed distribution, while RME samples showed greater clustering, with the two groups generally distinguishable in ordination space (Fig. 5a). Environmental factor fitting revealed that TC and TOC oriented toward the CAP1 positive direction, showing positive correlations with functional gene composition; TN and ORP oriented toward the CAP1 negative direction, exhibiting negative correlations; and pH oriented toward the CAP2 negative direction. Mantel test results were consistent: in CRCE, TC (*P* < 0.01) and TN (*P* < 0.01) showed highly significant correlations with functional genes, while pH (*P* < 0.05) and TOC (*P* < 0.05) exhibited significant correlations, with TC, TOC, and TN showing positive correlations and pH negative correlations. In RME, correlations between environmental factors and functional genes were generally weaker, and correlation patterns differed from those in CRCE (Fig. 5b).

## Discussion

### Coculture reshapes the soil physicochemical environment to enhance soil fertility

Thirteen months of continuous monitoring revealed that crayfish-plant coculture does not merely alter a single physicochemical indicator of the soil but rather enhances soil fertility and environmental stability through two synergistic pathways: carbon enrichment and redox homeostasis. The significantly higher TC and TOC contents in CRCE, coupled with lower intra-annual variability compared to RME, directly demonstrate the amplifying effect of the coculture model on the soil organic carbon pool—a fundamental component of soil fertility. This finding is highly consistent with that of Guo et al. (2025), who reported that soil organic carbon content in 10–15-year rice-crayfish coculture fields was significantly increased by 31.8%–37.2% compared to rice monoculture, alongside enhanced aggregate stability, higher soil nutrient levels, and lower redox potential (Guo et al. 2025). While previous studies have documented carbon sequestration in rice-crayfish systems, the mechanistic links between environmental stabilization, community assembly processes, network topology, and functional gene repertoires have remained unexplored. By integrating long-term time-series analysis, quantitative assembly modeling, and functional metagenomics within a single framework, this study provides the first comprehensive mechanistic explanation for how coculture systems simultaneously achieve soil fertility enhancement and microbial community stability. This observed carbon sequestration aligns with a growing body of evidence highlighting the model's potential to increase soil organic carbon stocks, particularly through the formation of water-stable macroaggregates that provide physical protection for organic carbon (Chen et al. 2025). This effect aligns closely with the functional role of *Procambarus clarkii* as an ecosystem engineer: its burrowing behavior alters the physical structure of the soil, its detritivorous feeding continuously introduces exogenous organic matter (residual bait, feces, decaying macrophytes), and its bioturbation promotes the formation of organo-mineral complexes, specifically, bioturbation increases the contact between organic matter and mineral surfaces, particularly iron and aluminum oxides, which are abundant in these subtropical paddy soils (Six et al. 2002). The formation of such complexes—through ligand exchange, polyvalent cation bridging, and hydrophobic interactions—physically protects organic carbon from enzymatic attack and microbial mineralization (Lehmann et al. 2020), thereby slowing the rapid mineralization of organic carbon (Li et al. 2025). As proposed in previous studies, these biotic activities act as key drivers reshaping the biogeochemical cycles that underpin the system's ecological sustainability (Zhu et al. 2023). Concomitantly, the CRCE system maintained consistently low redox potentials (− 150 to − 50 mV) throughout the monitoring period, whereas RME exhibited drastic fluctuations from strongly oxidizing (> 200 mV) to strongly reducing (< − 100 mV) conditions. Guo et al. (2025) similarly reported that long-term rice-crayfish coculture significantly lowers soil ORP. This observation aligns with our findings, collectively pointing to the formation of a well-buffered redox microenvironment in coculture soils—a mechanism likely driven by a dynamic equilibrium between oxygen consumption (fueled by organic matter mineralization) and microscale oxygen influx (mediated by crayfish burrowing activity and rice root aerenchyma; Hu et al. 2016). This stable reducing environment not only facilitates the preservation of soil organic carbon but also, via sustained carbon input, supplies continuous substrates for oxygen-consuming metabolic processes. This well-buffered redox microenvironment has profound implications for microbial metabolism. The coexistence of reducing bulk soil and oxidized microsites creates spatial gradients in electron acceptor availability, enabling the simultaneous occurrence of aerobic and anaerobic processes. Aerobic respiration and nitrification can proceed in oxygenated microzones (e.g., burrow walls, root surfaces), while anaerobic processes such as denitrification, iron reduction, and methanogenesis occur in the anoxic matrix (McAllister et al. 2021). This spatial coupling promotes metabolic complementarity and reduces competition for electron acceptors, as evidenced by the co-enrichment of aerobic nitrifiers (*Nitrospira*) and anaerobic iron reducers (*Geobacter*) in CRCE soils. Together, these interconnected processes maintain the foundational soil fertility and redox homeostasis that regulate subsequent microbial community assembly and dynamics. Our findings thus demonstrate that coculture enhances soil fertility not through a singular pathway but via the integrated modulation of carbon sequestration and redox stabilization—two synergistic processes that underpin the system’s functional resilience.

### Coculture drives functional differentiation in soil microbial community structure and keystone taxa

The reshaping of the soil physicochemical environment inevitably leads to responses in the microbial community, directly influencing community compositional stability. 16S rRNA gene amplicon sequencing revealed significant compositional divergence among the three systems. RME showed anomalous enrichment of Firmicutes (peak abundance 45.9%), a phylum containing numerous fast-growing facultative anaerobes often associated with pulsed inputs of labile organic matter (e.g., straw return) and drastic redox fluctuations (Hartmann et al. 2015). In stark contrast, coculture systems (CRCE and CWCE) exhibited significantly lower Firmicutes abundance, while Proteobacteria, Bacteroidetes, and Chloroflexi constituted stable core microbial taxa. Wang et al. (2023) also identified these as dominant phyla in soils of rice-crayfish coculture systems (Wang et al. 2023). This shift in community structure directly corresponds with the persistently low and stable ORP and increased total carbon observed in coculture soils, demonstrating that environmental stabilization translates into microbial community stabilization.

More notably, the coculture systems specifically enriched a suite of keystone taxa possessing crucial ecological functions for soil fertility. The relative abundances of *Geobacter*, *Dechloromonas*, and *Sulfuricurvum* were significantly higher in CRCE compared to RME. *Geobacter* are renowned iron reducers, coupling the anaerobic oxidation of organic matter with the reduction of iron oxides and mediating extracellular electron transfer (Lovley et al. 2004); *Dechloromonas* possesses capabilities for dissimilatory iron reduction, denitrification, and aromatic hydrocarbon degradation (Achenbach et al. 2001; Duffner et al. 2021); *Sulfuricurvum* are sulfur-oxidizing autotrophs preferring microoxic niches (Cron et al. 2019). The synergistic enrichment of these groups in CRCE suggests the formation of an "iron‑nitrogen‑sulfur" coupled metabolic network within the coculture soil—a network capable of efficiently mineralizing organic matter while buffering redox fluctuations, representing a direct manifestation of functional stability at the microbial level. This observation provides empirical support for the proposed microbial-driven biogeochemical cycling that characterizes these integrated systems (Zhu et al. 2023). Conversely, the significantly enriched taxa in RME, such as *Pseudomonas*, *Stenotrophomonas*, and *Flavobacterium*, are predominantly opportunistic r-strategists that proliferate rapidly after disturbance; their dominance corroborates the instability of the monoculture system and its limited capacity for sustained fertility regulation.

### Coculture enhances complexity of microbial interaction networks

Compositional differences in communities represent only a superficial manifestation of microbial response; deeper changes lie in the reorganization of interspecific interactions that underpin community functional stability. Co-occurrence network analysis showed that CRCE constructed a highly complex interaction network (1,625 nodes, 7,986 edges), with a remarkably high proportion (98.6%) of positive interactions. A high percentage of positive associations typically indicates extensive niche sharing, cross-feeding (syntrophy), or co-metabolism (Faust and Raes 2012), suggesting that microbial taxa in CRCE have formed highly cooperative metabolic alliances that enhance system-level functional efficiency. This finding aligns with research on long-term integrated polyculture systems (e.g., rice-crayfish-turtle), which demonstrated that such practices can shape more complex and stable microbial community network structures, ultimately contributing to enhanced soil health and crop productivity through soil-microbe interactions (Li et al. 2022). Zhang et al. (2024) similarly found that the rice-crayfish farming model significantly increased network complexity in both leftsoil and subsoil, thereby enhancing network stability (Zhang et al. 2024). Although the CWCE network was smaller (694 nodes), it exhibited a significantly higher proportion of negative interactions (16.1%), which might promote functional differentiation and maintain community diversity in resource-limited environments.

Unexpectedly, the RME network possessed the highest number of edges (20,684) and an almost entirely positive association (97.8%), appearing highly cooperative from a topological perspective. However, considering the absolute dominance of opportunistic taxa (e.g., *Pseudomonas*, *Bacillus*) in RME, this dense positive association more likely reflects functional redundancy rather than true cooperation—i.e., numerous species occupy similar niches, rendering the system's buffering capacity against external disturbances inherently fragile (Loreau et al. 2001). Liu et al. (2024) also noted in their study of rice-fish coculture systems that while traditional rice-fish coculture increased microbial interaction strength, its impact on network stability remained complex (Liu et al. 2024). Our findings demonstrate that network complexity alone is not a reliable stability indicator; instead, a moderately complex network structure incorporating functionally differentiated keystone taxa (as exhibited by CRCE) represents a more robust architecture for maintaining community compositional and functional stability—a key requirement for sustained soil fertility. The superior performance of CRCE over CWCE in regulating soil fertility and community stability may be attributed to differences in plant type. Rice, as a C3 plant, produces root exudates rich in organic acids and sugars that may more effectively promote the growth of specific functional taxa such as *Geobacter*. In contrast, waterweed (*Elodea nuttallii*), as a submerged macrophyte, likely has different carbon input patterns and rhizosphere effects (Pang and Xu 2024). Additionally, the well-developed aerenchyma of rice roots provides more efficient micro-scale oxygenation, potentially reinforcing the 'macro-reducing, micro-oxidizing' spatial pattern that favors the enrichment of functionally coupled keystone taxa. These plant-specific differences highlight the importance of plant selection in optimizing coculture system design.

### Environmental filtering and ecological processes jointly shape microbial community assembly in coculture systems

Microbial community assembly is not a stochastic event; the relative contributions of deterministic and stochastic processes in different habitats determine community structure stability and functional trajectory. Null model analysis based on iCAMP revealed that drift and homogeneous selection were the dominant forces shaping community assembly in CRCE (accounting for 40%–60% and 30%–50%, respectively). The high proportion of homogeneous selection implies a strong and consistent environmental screening pressure within the coculture soil. In striking contrast, the contribution of drift in RME was as high as 70%–90%, while homogeneous selection accounted for only 10%–20%, indicating that monoculture soils lack consistent environmental filtering, and community succession is predominantly governed by stochastic birth/death/immigration events (Zhou and Ning 2017). This assembly pattern of "weakened environmental selection—stochastic dominance" is mutually reinforcing with the drastic ORP fluctuations, carbon pool instability, and frequent outbreaks of opportunistic taxa observed in RME, explaining the system's inability to maintain stable community composition and fertility-regulating capacity.

Environmental factor–microbe association analysis further elucidated the core drivers of community organization in each habitat. In CRCE, NO₃⁻-N and NO₂⁻-N jointly explained 36.3% of community variation and showed significant positive correlations with diversity and key nitrifying taxa (e.g., Nitrospira, Planctomycetes), suggesting that active nitrification within the stable redox environment is a key organizer of community structure linked to nitrogen fertility. In CWCE, TN, TP, and NH₄⁺-N were the primary drivers (20.3%), reflecting stronger nutrient retention pressures within the macrophyte system. In RME, pH and TN jointly dominated (29.9%), confirming that pH fluctuations and nitrogen pulses impose strong but inconsistent disturbances. These results clearly demonstrate that coculture systems, by stabilizing key environmental gradients, shift community assembly from “stochastic drift dominance” to “deterministic homogeneous selection dominance” —a core ecological mechanism underpinning the stability of both microbial community composition and its fertility-regulating functions. This deterministically dominated assembly mode implies greater predictability of system functions, providing a theoretical basis for targeted regulation of microbial communities. By maintaining stable redox conditions and consistent carbon inputs, farmers can exert 'left-down' control on microbial communities, steering them toward desired functional states—for example, enhancing nitrification capacity to improve nitrogen use efficiency, or promoting iron-reducing bacteria to stabilize organic carbon.

### Coculture restructures the functional potential of soil microbial communities

Our metagenomic analysis revealed that the transition from RME to CRCE significantly restructured the functional gene composition of soil microbial communities, as evidenced by the significant separation between CRCE and RME samples in beta diversity analysis (Adonis R^2^ = 0.168, *P* = 0.001). This functional divergence occurred despite similar functional gene evenness (Shannon index) between the two systems, suggesting that crayfish introduction selectively enriches or suppresses specific microbial functions rather than broadly altering community functional diversity. The significantly lower Chao1 index in CRCE soils (*P* = 0.020) indicates reduced functional gene richness, which may reflect the selective pressure imposed by the distinct physicochemical environment created by crayfish bioturbation. Hou et al. similarly reported that integrated rice-crayfish farming significantly impacted soil bacterial communities, with total nitrogen and total phosphorus emerging as primary factors contributing to community alterations (Hou et al. 2024). These findings align with previous observations that aquaculture model-specific biogeochemical cycling patterns emerge under coculture conditions.

The significant enrichment of *pps*/*ppsA*, *PGK*/*pgk*, and *E2.2.1.1*/*tktA*/*tktB* in CRCE soils indicates enhanced genetic potential for carbon fixation and central carbon metabolism under coculture conditions. The *pps*/*ppsA* gene cluster, involved in gluconeogenesis and anaerobic CO₂ fixation, suggests that CRCE microbiomes may possess enhanced capacity to incorporate inorganic carbon into organic forms. This observation aligns with the significantly higher total organic carbon content documented in CRCE soils and is consistent with evidence that rice-crayfish systems enhance soil organic carbon stocks through sustained exogenous organic inputs and physical protection within stable aggregates. The concurrent enrichment of *PGK*/*pgk* (glycolysis/gluconeogenesis) and *tktA*/*tktB* (pentose phosphate pathway) suggests flexible central carbon metabolism capable of responding to variable carbon substrates and redox conditions imposed by crayfish bioturbation. In contrast, RME soils exhibited pronounced enrichment of genes associated with complete organic matter oxidation and respiratory energy conservation. The significant enrichment of *acnA* (aconitase), *sdhA*/*frdA* and *sdhB*/*frdB* (succinate dehydrogenase/fumarate reductase) indicates enhanced TCA cycle activity in monoculture soils, suggesting greater reliance on complete mineralization of organic substrates. The exceptionally high enrichment of *E5.4.99.2A*/*mcmA1* (methylmalonyl-CoA mutase) in RME, the most pronounced differential expression observed, suggests that monoculture microbiomes may rely more extensively on lipid-derived carbon sources, possibly reflecting cumulative effects of annual straw incorporation. The enrichment of *accC (*acetyl-CoA carboxylase) in RME further supports enhanced fatty acid biosynthesis capacity. The enrichment of anaerobic metabolism genes, *por*/*nifJ* and *korB*/*oorB*/*oforB*, in RME indicates that monoculture soils maintain genetic potential for anaerobic carbon transformations despite fluctuating redox conditions (Chen et al. 2025). Collectively, these patterns indicate that crayfish introduction shifts microbial carbon processing from complete aerobic oxidation toward more flexible metabolic strategies incorporating enhanced carbon fixation potential—a functional reorganization that parallels the observed enhancement of soil organic carbon stocks in coculture systems.

The differential enrichment of nitrogen cycle genes between CRCE and RME provides evidence for pathway-specific restructuring of nitrogen transformation potentials. The significant enrichment of *hao* (hydroxylamine oxidoreductase) in CRCE indicates enhanced nitrification potential under coculture conditions, likely reflecting the creation of aerobic microsites through crayfish burrowing activity and rice root aerenchyma (Hu et al. 2016). This aligns with environmental factor analysis, which identified NO₃⁻-N and NO₂⁻-N as key drivers of community variation in CRCE.

The significant enrichment of *norC* (nitric oxide reductase) in CRCE suggests potential for enhanced denitrification intermediate processing. Most notably, the enrichment of *nasB* (assimilatory nitrate reductase) indicates enhanced capacity for nitrate assimilation into organic nitrogen. This gene-level evidence directly supports the proposition that coculture systems promote nitrogen retention through microbial biomass incorporation rather than denitrification losses (Chang et al. 2025). The enrichment of *nasB*, coupled with the absence of significant enrichment for dissimilatory nitrate reduction genes, suggests that CRCE microbiomes preferentially channel inorganic nitrogen toward organic nitrogen pools—a mechanism contributing to nitrogen fertility maintenance while minimizing pollution risks. This finding provides direct gene-level evidence supporting the notion that coculture systems are advantageous for nitrogen assimilation and pollutant removal (Zhu et al. 2023). In contrast, RME soils exhibited significant enrichment of denitrification and dissimilatory nitrate reduction genes. The enrichment of *nirK* (nitrite reductase), *narB* (nitrate reductase), and *nirA* (assimilatory nitrite reductase) suggests enhanced potential for denitrification and dissimilatory pathways that may increase gaseous nitrogen losses while responding to pulsed nitrogen inputs from fertilization. Collectively, our results validate the proposed hierarchical causal pathway: bioturbation-induced environmental stabilization drives deterministic community assembly, which promotes keystone taxon enrichment and functional integration, ultimately regulating soil fertility and maintaining community stability.

## Conclusions

This 13-month field study demonstrates that crayfish-rice coculture fundamentally reshapes the soil microbiome into a more stable, functionally integrated state, thereby regulating soil fertility while sustaining both compositional and functional stability of the microbial community. By integrating time-series analysis, network ecology, quantitative community assembly modeling, and functional metagenomics, we establish a hierarchical causal pathway: crayfish bioturbation and plant presence synergistically stabilize the physicochemical environment via carbon sequestration and redox homeostasis, which in turn imposes strong deterministic selective pressures (dominance of homogeneous selection). This selection enriches functionally coupled keystone taxa (e.g., *Geobacter*, *Sulfuricurvum*, *Nitrospira*) and fosters cooperative ecological networks characterized by positive interactions. Ultimately, this manifests as an enriched and integrated functional gene repertoire supporting coupled carbon, nitrogen, phosphorus, and sulfur biogeochemical cycling, directly underpinning enhanced soil fertility. In contrast, rice monoculture soils are governed by stochastic drift, dominated by opportunistic taxa, and exhibit mere functional redundancy rather than true functional stability. Our findings provide a mechanistic framework explaining how anthropogenically designed coculture systems achieve soil microbiome stability and targeted fertility regulation, advancing the understanding of sustainable agriculture from macroscopic observations to microbial mechanisms. Furthermore, this work offers actionable insights, for example, management practices such as regulating the carbon-to-nitrogen ratio in organic amendments or optimizing flooding depth to maintain stable redox conditions could strengthen deterministic selection and directionally enrich functional taxa, providing practical strategies for optimizing soil health in integrated farming systems, highlighting the value of harnessing microbial processes to optimize ecological intensification.

## Supplementary Information

Supplementary Material 1.

## Data Availability

The data have been deposited in the NCBI Sequence Read Archive under BioProject ID PRJNA1435542.
