# Supplementary material for: The crayfish-rice coculture model contributes to regulating the soil fertility of rice fields and maintaining the stability of soil microbial community composition and function
Source: Adv Biotechnol (Singap). 2026 Apr 28;4(2):18. doi: 10.1007/s44307-026-00106-x (PMC13121677; doi:10.1007/s44307-026-00106-x)
Supplement: Supplementary file 1 — Supplementary Material 1. [file 44307_2026_106_MOESM1_ESM.docx]

**Advanced Biotechnology**

Supplemental information

**The crayfish-rice coculture model contributes to regulating the soil fertility of rice fields and maintaining the stability of soil microbial community composition and function**

Dongdong Wei^1,3^, Chengguang Xing^1^, Shenzheng Zeng^1^, Dongwei Hou^1^, Zhixuan Deng^1^, Xinghai Long^1^, Hao Wang^1^, Renjun Zhou^1^, Lingfei Yu^1^, Nana Shu^4^, Zhonghu Tao^4^, Xi Zhou^4^, Shaoping Weng^2^, Jianguo He^1, 2*^, Zhijian Huang^1*^

^1^State Key Laboratory of Biocontrol, Southern Marine Sciences and Engineering Guangdong Laboratory (Zhuhai), School of Agriculture and Biotechnology, Sun Yat-sen University, Shenzhen, China

^2^School of Life Sciences, Sun Yat-sen University, Guangzhou, China

^3^Guangxi Academy of Marine Sciences, Guangxi Academy of Sciences, Nanning, China

^4^ Qianjiang Crayfish Industry Development and Promotion Center，Qianjiang, China

*Corresponding Authors: Jianguo He, lsshjg@mail.sysu.edu.cn

Zhijian Huang, lsshzhj@mail.sysu.edu.cn

**Fig. S1 Host tracking analysis of carbon cycling genes: linking functional potential to microbial taxa at the genus level**

**Fig. S2 Co-occurrence network analysis of carbon and nitrogen cycling functional genes in CRCE and RME soils**


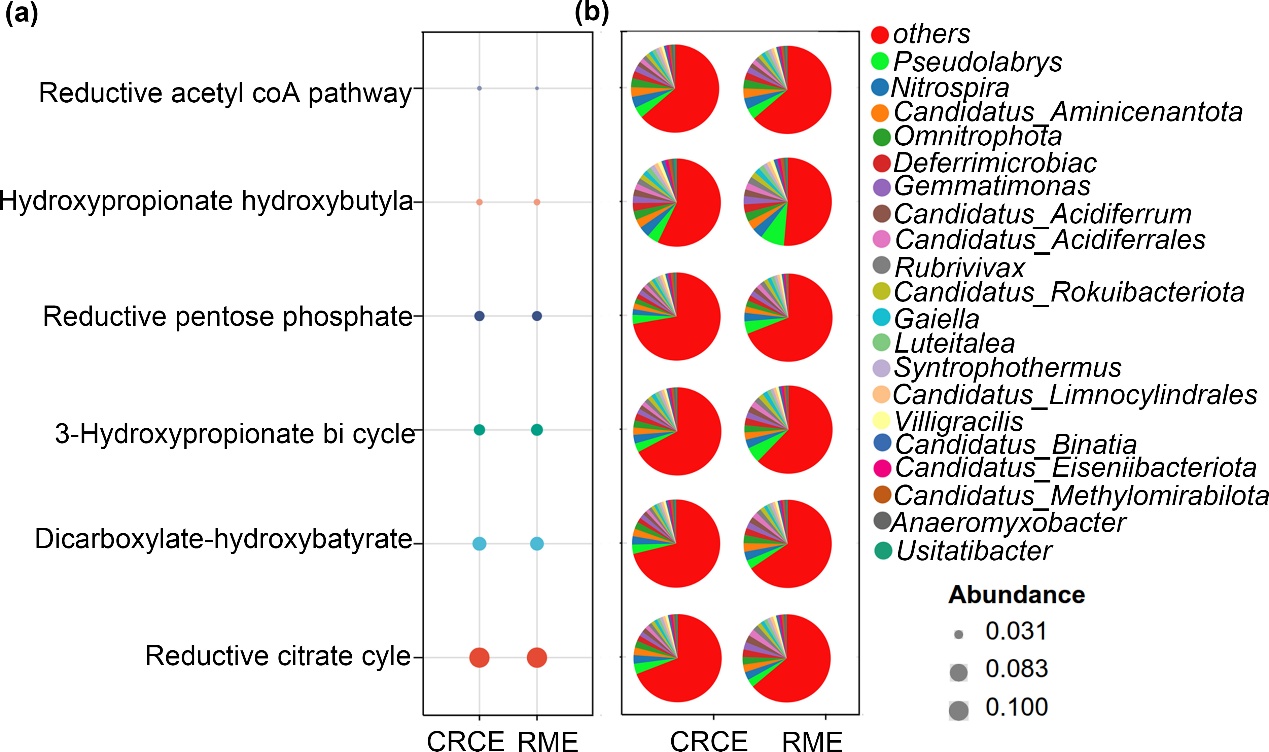


**Fig. S1 Host tracking analysis of carbon cycling genes: linking functional potential to microbial taxa at the genus level.** (**a**) Bubble plot showing the relative abundance of genes involved in six major carbon fixation pathways carried by key microbial genera in CRCE and RME soils. (**b**) Pie charts displaying the relative abundance of the corresponding microbial genera identified as hosts of carbon cycling genes in CRCE and RME soils, revealing the taxonomic distribution of functional potential.


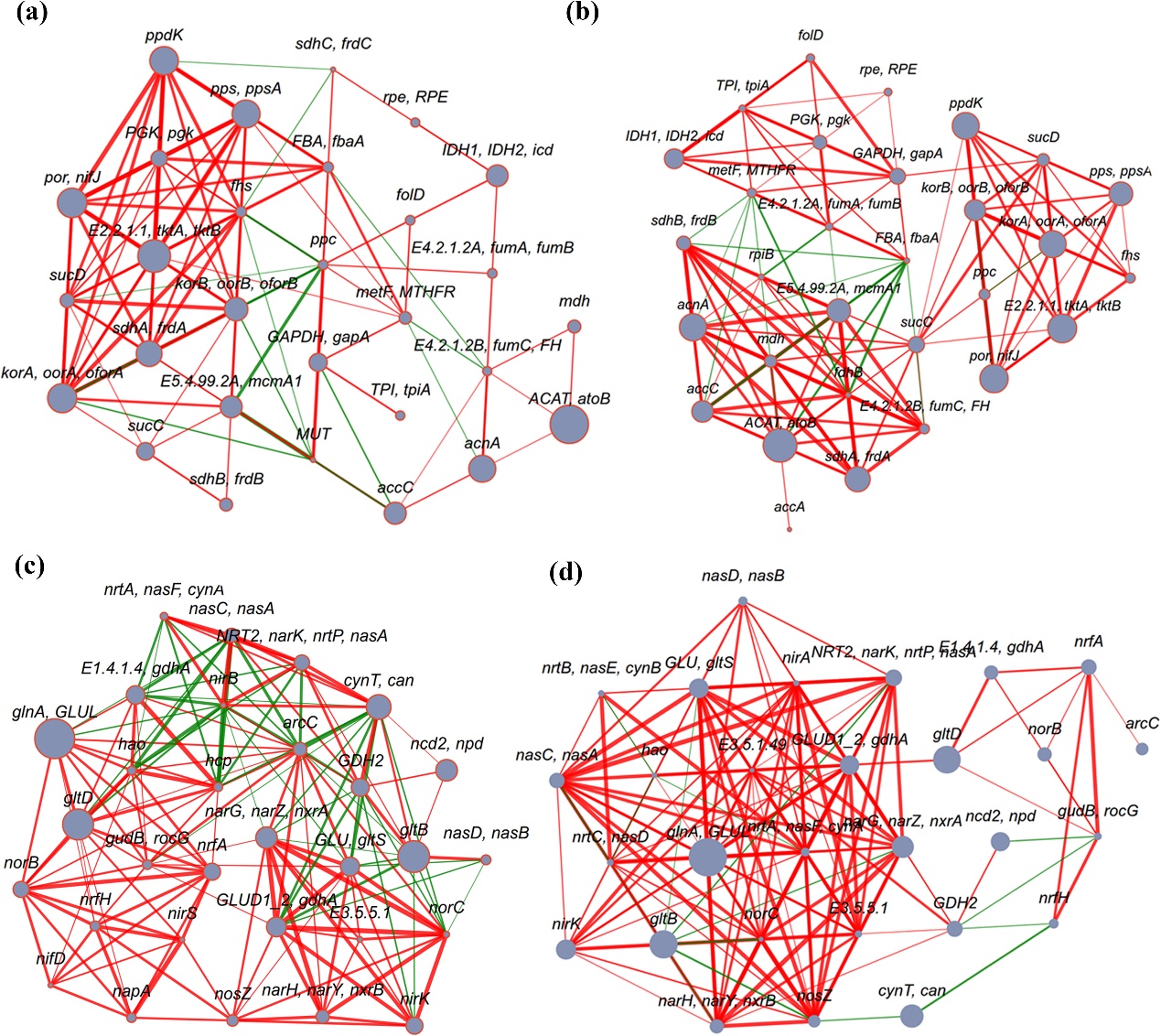


**Fig. S2 Co-occurrence network analysis of carbon and nitrogen cycling functional genes in CRCE and RME soils.** Networks were constructed based on Spearman correlation analysis. Panels (**a**) and (**b**) show carbon cycling functional gene networks in CRCE and RME soils, respectively. Panels (**c**) and (**d**) show nitrogen cycling functional gene networks in CRCE and RME soils, respectively.
